# Genetically Predicted Circulating Levels of Cytokines and the Risk of Cancer

**DOI:** 10.3389/fimmu.2022.886144

**Published:** 2022-07-05

**Authors:** Jie Song, Aole Li, Yu Qian, Bin Liu, Linshuoshuo Lv, Ding Ye, Xiaohui Sun, Yingying Mao

**Affiliations:** ^1^ School of Public Health, Zhejiang Chinese Medical University, Hangzhou, China; ^2^ The Fourth College of Clinical Medicine, Zhejiang Chinese Medical University, Hangzhou, China; ^3^ School of Life Sciences, Westlake University, Hangzhou, China

**Keywords:** mendelian randomization, inflammation, cytokines, cancer, single nucleotide polymorphism

## Abstract

**Background:**

Inflammation plays a pivotal role in the pathogenesis of cancer. Though previous studies have reported a link between several inflammatory biomarkers and risk of certain types of cancer, there is a lack of systematic investigation. Therefore, we aimed to assess the role of circulating cytokines on the risk of cancer using a two-sample Mendelian randomization (MR) approach.

**Method:**

We used genetic variants associated with circulating levels of cytokines from a meta-analysis of genome-wide association studies (GWASs) of 8,293 Finns as instrumental variables. Summary level data of 20 site-specific cancer were obtained from the UK BioBank including up to 456,348 participants of European ancestry. We performed two-sample MR analyses using inverse-variance weighted (IVW) method as the main method, followed by weighted-median and likelihood-based methods as sensitivity analysis. Pleiotropic and outlier variants were assessed by MR-Egger regression and MR Pleiotropy RESidual Sum and Outlier (MR-PRESSO) test.

**Results:**

224 genetic variants associated with 27 circulating cytokines achieving genome-wide significance (*P*<5×10^-8^) were used as IVs. After Bonferroni correction, genetically predicted high levels of interleukin-18 (IL-18) were associated with a decreased risk of acute myeloid leukemia (odds ratio (OR) per 1 standard deviation (SD) increase = 0.55, 95% confidence interval (CI):0.43-0.69, *P*=5.39×10^-7^), and circulating levels of IL-17 were associated with altered stomach cancer risk (OR per 1 SD increase = 0.15, 95% CI: 0.07-0.36, *P*=1.25×10^-5^) by IVW. Results were stable across sensitivity analyses, and MR-Egger regression did not suggest the presence of directional pleiotropy. Additionally, we found suggestive evidence for 48 cytokine-cancer associations including tumor necrosis factor related apoptosis-inducing ligand (TRAIL) and cutaneous T-cell attracting chemokine (CTACK) with the risk of several types of cancer (9.26×10^-5^≤*P*<0.05).

**Conclusions:**

By using a genetic epidemiological approach, our study systematically evaluated the role of circulating cytokines on the risk of cancer, and provided clues for potential therapeutic targets. However, the exact underlying biological mechanism warrants further investigation.

## Introduction

Cancer is a leading cause of death before the age of 70 years in 183 countries in 2019 (1). Globally, an estimated 19.3 million new cancer cases and approximately 10.0 million cancer deaths occurred in 2020 (2). Established risk factors for cancer include obesity, smoking, alcohol consumption, and infection (3–7). Inflammation has been demonstrated to play a pivotal role in carcinogenesis, since the reactive oxygen/nitrogen species from inflammation damage not only DNA but also other biomacromolecules, such as proteins and lipids, may result in their dysfunction, thus exerting cancer promoting effects (8). Previous studies have shown that NLRP3 inflammasome and cytosolic multi-protein complexes involved in innate immune response (9), can promote the development of several malignancies, including head and neck squamous cell carcinoma, fibrosarcoma, melanoma, stomach cancer and lung cancer (10–15). Observational data also indicated that chronic inflammation can increase the risk of certain types of cancer. For example, chronic prostatitis can increase the risk of prostate cancer by 2-3 times, and ulcerative colitis can increase the risk of colon cancer by 6-19 times (16). In addition, several inflammatory markers have been tested in relation to cancer incidence. For instance, a meta-analysis showed that higher concentrations of circulating C-reactive protein (CRP), a non-specific marker of systemic inflammation, were associated with a higher risk of breast cancer (hazard ratio (HR)=1.14, 95% confidence interval (CI) 1.01-1.28), lung cancer (HR=2.03, 95% CI 1.59-2.60), colorectal cancer (OR=1.34, 95% CI 1.11-1.60) and prostate cancer (HR=1.09, 95% CI 1.03-1.15) (17). These findings suggested that cytokines may play pivotal roles in carcinogenesis, and intervening on these inflammatory biomarkers in cancer may help prevent its incidence and aid in the development of novel therapeutic targets (17).

Mendelian Randomization (MR) utilizes genetic variants as instrumental variables (IVs) to estimate the potential causal relationship between the exposure (i.e., circulating cytokines) and the outcome (i.e., cancer risk) (18). Since genotypes are randomly distributed in the process of gamete formation, the causal inference from MR analyses are less susceptible to common confounding factors in conventional observational studies, such as postnatal environment, socio-economic status, and behavioral factors (19). Moreover, since genotypes preceded the onset of diseases, MR analyses are less prone to reverse causation in traditional observational studies. Therefore, it becomes widely used to assess the potential causal associations between exposures and diseases.

In the present study, by leveraging large-scale data of genome-wide association studies (GWAS) from the UK BioBank, we implemented a two-sample MR design to systematically investigate the role of circulating cytokines on the risk of different cancer types.

## Method

### Study Design and Data Source

**Figure 1:**
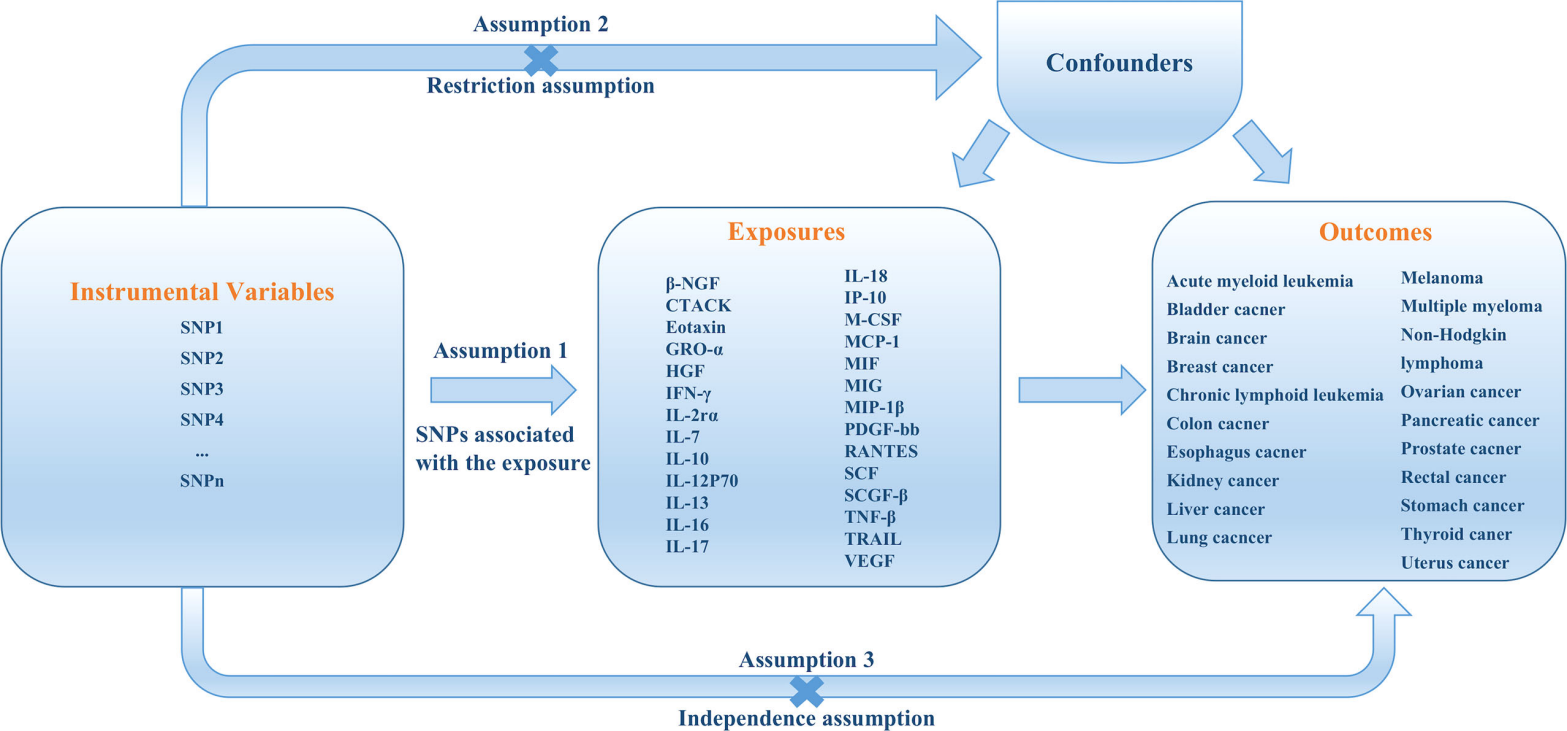
An overall design of the present study.

The overall design of the present study is shown in **Figure 1**. The detailed information on the summary-level data of GWASs on cytokines and 20 site-specific cancer from the UK BioBank are summarized in **Table S1**. Briefly, all data in our study were based on participants of European ancestry. The GWAS meta-analysis of cytokines included 8,293 Finnish individuals from three independent population cohorts: the Cardiovascular Risk in Young Finns Study, FINRISK1997, and FINRISK2002 Study (20). The effect estimates of each genetic variants retrieved were calculated using a additive genetic model and adjusted for the first ten genetic principal components, age, sex, and body mass index.

Summary-level data for the association between genetic variants and 20 site-specific cancer were obtained from the UK BioBank (UKBB), which is a large-scale cohort study of around 500,000 adults aged 40 to 69 years across the UK from 2006 to 2010 (21). In the present analyses, we included genetic data of up to 456,348 participants from UKBB (**Table S1**).

### Selection of Genetic Variants

Single nucleotide polymorphisms (SNPs) associated with circulating levels of cytokines were selected at the genome-wide significance level (*P*<5×10^-8^). We then pruned these SNPs in linkage disequilibrium (LD) using an r^2^ threshold < 0.1, and selected the SNPs with the lowest *P*-value. A total of 958 independent SNPs were selected as IVs. Among them, 129 SNPs were associated with more than one cytokine and were thus excluded. Among the remaining 829 SNPs, 605 were not available in the outcome datasets. Finally, 224 SNPs associated with circulating levels of 27 cytokines were included in the subsequent MR analyses. Detailed information of the 27 cytokines and the SNPs used as IVs are displayed in **Table S2**.

### Statistical Analysis

First, we calculated F-statistics to quantify the strength of the IVs, with the equation of *F*=*R* ^2^×(*N*−1−*k*)/(1−*R* ^2^), in which R^2^ represents the variance explained by the IVs, N indicates the sample size, and k is the number of SNPs included in the instrument (22).

We then used inverse-variance-weighted (IVW) method as the main MR analysis to evaluate the potential causal effects of the 27 cytokines on the risk of 20 site-specific cancer (19, 23). Cocrane’s Q test was applied to assess the heterogeneity between the SNPs, and a fixed-effects model was used when there was no evidence of heterogeneity; otherwise, a random-effects model was used. In addition, to assess the robustness of our main findings, we used a series of sensitivity analyses, including the weighted-median and likelihood-based methods. Specifically, the weighted-median method combines the unweighted or weighted estimation with the median. As long as the weight of the causal effect calculated by the effective instrumental variable reaches 50%, a consistent estimation of the causal effect can be obtained (22). Meanwhile, the likelihood-based method evaluates the potential causal relationship under the assumption of a linear association between the risk factor and the outcome variables (24). Moreover, MR-Egger regression was performed to assess the potential directional pleiotropy. The slope of MR-Egger regression can suggest pleiotropy corrected causal estimates, and the value of the intercept can provide an estimate of the degree of pleiotropy (25). The Mendelian randomization pleiotropy residual sum and outlier (MR-PRESSO) test was also used to detect and correct for horizontal pleiotropic outliers. It conducts a global test of heterogeneity by regressing the SNP-outcome associations on the SNP-exposure associations and comparing the observed distance of each SNP from the regression with the distance expected under the null hypothesis of no pleiotropy (26).

Furthermore, to minimize the influence of pleiotropic instruments on MR estimates, we manually scanned the SNPs used as IVs in the GWAS Catalog, and excluded those associated with secondary phenotypes at genome-wide significance. We then reran the MR analyses using the updated IVs. Additionally, to test for the stability of our findings, we performed “leave-one-out” analyses, which excluded one single SNP at a time, and re-run the MR analysis using IVW method with the remaining IVs.

All analyses were performed using R (version 3.6.0) and related R packages (MendelianRandomization and MR-PRESSO). Associations with *P* values<9.26×10^-5^ (*P* = 0.05/27 cytokines/20 site-specific cancers) were considered statistically significant after Bonferroni correction for 27 cytokines and 20 site-specific cancer. A *P*-value < 0.05, but above the Bonferroni correction threshold, was considered suggestive evidence for a potential causal association.

## Results

**Table S3** presents the detailed information of the IVs used for circulating levels of the 27 cytokines and growth factors. The median of F-statistics ranged from 30 to 788.96, satisfying the threshold of >10, suggesting that the IVs used in our study were unlikely to suffer from weak instrument bias (27).

**Figure 2:**
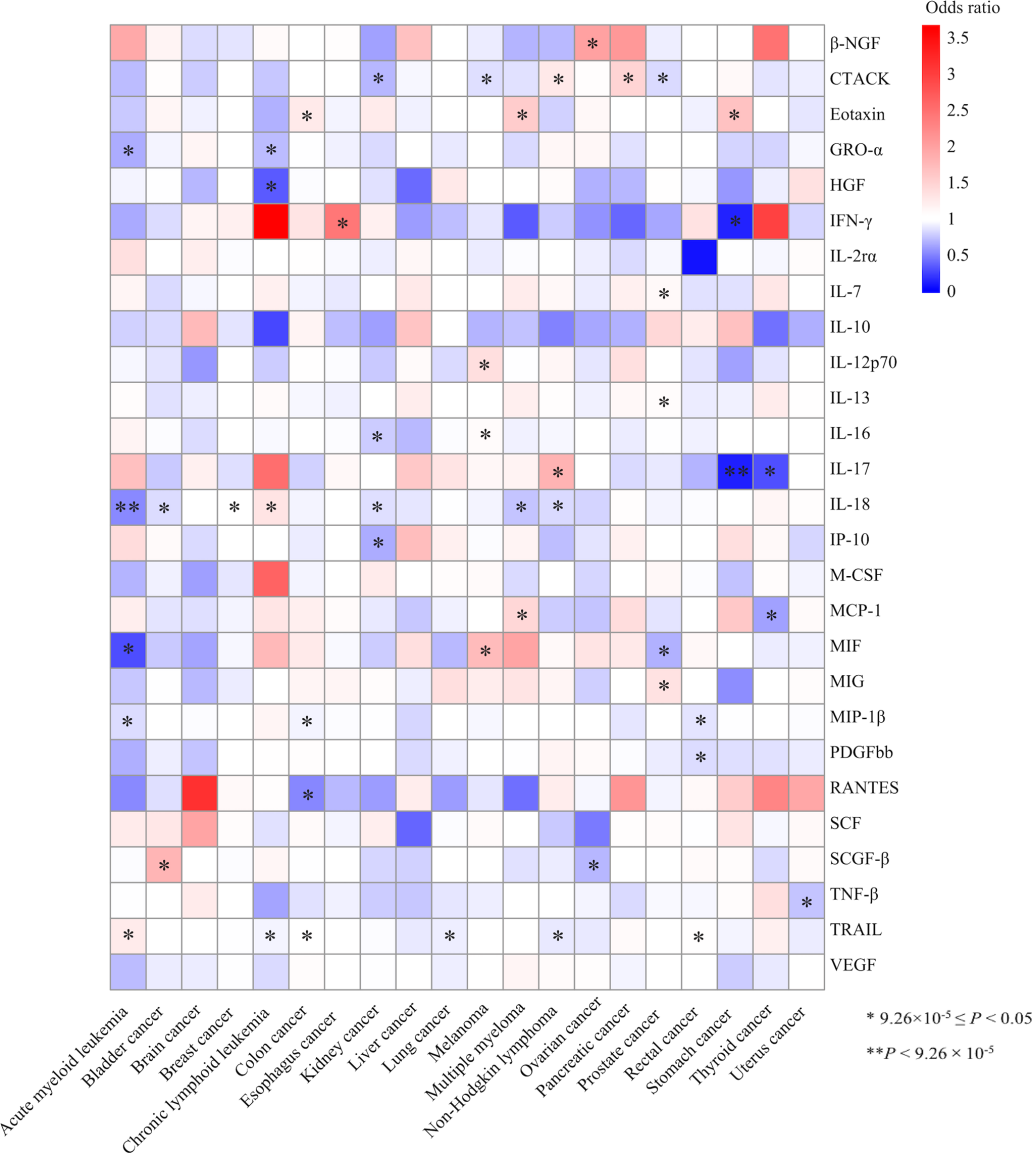
Heatmap of the associations of the 27 cytokines with the risk of 20 site-specific cancer from the inverse variance weighted (IVW) method. One asterisk indicates the suggestive evidence for a potential causal association (9.26×10^-5^≤ *P <*0.05), while two asterisks denote that the associations were statistically significant after multiple comparison correction (*P*<9.26×10^-5^). Colour is scaled based on the odd ratio (OR) of the MR association estimate.

The MR association estimates of the 27 cytokines with the risk of 20-site specific cancer are shown in **Figure 2**. Specifically, genetically predicted higher levels of circulating IL-18 was associated with a decreased risk of acute myeloid leukemia (OR=0.55, 95% CI: 0.43-0.69, *P*=5.39×10^-7^, per 1 standard deviation (SD) increase). Detailed information of the IVs used for circulating IL-18 levels and their associations with the risk of acute myeloid leukemia (AML) are presented in **Table 1**. In sensitivity analysis using different MR methods, the association remained statistically significant and the effect estimates were similar (OR=0.44, 95% CI: 0.32-0.60, *P*=1.23×10^-7^ for the weighted-median method; OR=0.55, 95% CI: 0.44-0.70, *P*=1.06×10^-6^ for the maximum-likelihood method). Moreover, there was no evidence for the presence of directional pleiotropy (*P* for MR-Egger intercept =0.095; *P* for MR-PRESSO global test=0.381; and number of outlier SNPs=0), and the results from MR-PRESSO test was similar (OR=0.55, 95% CI: 0.43-0.69, *P*=1.55×10^-4^). None of the SNPs used as IVs had documented pleiotropy as we searched the GWAS Catalog (last accessed on January 31, 2022). In the leave-one-out sensitivity analysis, the association estimates of genetically predicted IL-18 levels with the risk of AML did not change substantially after excluding one single SNP at a time (**Figure S1**).

**Table 1:** MR effect estimates for associations of circulating IL-18 with the risk of AML and IL-17 with stomach cancer.

	Chr	Position	Effect allele	OR (95% CI)	*P* for association	P for heterogeneity	P intercept from MR-Egger regression
IL-18 and AML							
rs61902693	2	31850035	G	0.82 (0.26, 2.60)	0.739		
rs144736172	2	31968142	T	0.49 (0.22, 1.09)	0.080		
rs71478720	2	32226986	G	0.76 (0.39, 1.49)	0.424		
rs71446036	2	32272399	G	0.37 (0.16, 0.87)	0.023		
rs6716179	2	32393076	C	0.51 (0.21, 1.26)	0.144		
rs212761	2	32429288	C	0.38 (0.17, 0.85)	0.018		
rs141091241	2	32466607	G	1.03 (0.33, 3.21)	0.964		
rs212724	2	32467489	C	0.44 (0.20, 0.93)	0.032		
rs385076	2	32489851	C	0.40 (0.20, 0.79)	0.009		
rs17229943	2	32659489	G	1.21 (0.39, 3.79)	0.739		
rs116656892	5	68186028	C	1.92 (0.73, 5.03)	0.185		
rs7601267	5	68535015	C	1.06 (0.33, 3.56)	0.029		
rs115267715	5	68682536	C	0.25 (0.04, 1.62)	0.144		
rs660558	11	112009605	C	0.27 (0.08, 0.89)	0.031		
rs2038534	11	112111460	C	0.94 (0.29, 3.10)	0.921		
rs212722	11	112250980	C	0.44 (0.20, 0.93)	0.032		
Inverse-variance weighted				0.55 (0.43, 0.69)	5.39×10^-7^	0.302	
Weighted median				0.44 (0.32, 0.60)	1.23×10^-7^		
Maximum-likelihood				0.55 (0.44, 0.70)	1.06×10^-6^		
MR-PRESSO				0.55 (0.43, 0.69)	1.55×10^-4^		
MR-Egger				/	/		0.095
IL-17 and stomach cancer							
rs2305051	3	122843212	C	0.14 (0.04, 0.49)	0.002		
rs1530455	3	122854899	C	0.17 (0.05, 0.52)	0.002		
Inverse-variance weighted				0.15 (0.07, 0.36)	1.25×10^-5^	0.816	
Maximum-likelihood				0.15 (0.06, 0.40)	1.07×10^-4^		

AML, acute myeloid leukemia; Chr, chromosome; CI, confidence interval; IL-17, interleukin-17; IL-18, interleukin-18; MR, Mendelian randomization; MR-PRESSO, Mendelian randomization pleiotropy residual sum and outlier; OR, odds ratio.

Meanwhile, we found an inverse association between circulating levels of IL-17 and the risk of stomach cancer using the IVW method (per 1 SD increase OR=0.15, 95% CI: 0.07-0.36, *P*=1.25×10^-5^, **Table 1**, **Figure 2**). One SNP, rs1530455, was found to be associated with other phenotypes at genome-wide significance level (**Table S4**). After excluding this SNP, the effect estimate did not change essentially (OR=0.14, 95% CI: 0.04-0.49, *P*=0.002).

In addition, we found 48 suggestive associations of a specific cytokine with the risk of site-specific cancer (9.26×10^-5^≤ *P <*0.05). The detailed results are shown in **Figure 2** and **Table S5**. Among them, IL-18 was nominally associated with the risk of six types of cancer, including bladder cancer (OR=0.86, 95% CI: 0.79-0.93, *P*=3.30×10^-4^), breast cancer (OR=1.05, 95% CI: 1.01-1.09, *P*=0.027), chronic lymphoid leukemia (CLL) (OR=1.39, 95% CI: 1.29-1.71, *P*=0.002), kidney cancer (OR=0.87, 95% CI: 0.77-0.99, *P*=0.028), multiple myeloma (OR=0.76, 95% CI: 0.63-0.90, *P*=0.002), non-Hodgkin lymphoma (OR=0.84, 95% CI: 0.74-0.96, *P*=0.008). Similarly, genetically determined circulating levels of tumor necrosis factor related apoptosis-inducing ligand (TRAIL) were nominally associated with the risk AML (OR=1.27, 95% CI: 1.06-1.52, *P*=0.011), colon cancer (OR=1.07, 95% CI: 1.01-1.13, *P*=0.021), CLL (OR=1.27, 95% CI: 1.06-1.52, *P*=0.011), lung cancer (OR=0.93, 95% CI: 0.87-0.99, *P*=0.034), non-Hodgkin lymphoma (OR=0.91, 95% CI: 0.84-0.99, *P*=0.034), and rectal cancer (OR=1.08, 95% CI: 1.01-1.15, *P*=0.032). Moreover, circulating cutaneous T-cell attracting chemokine (CTACK) levels were nominally associated with the risk of kidney cancer (OR=0.72, 95% CI: 0.64-0.85, *P*=1.64×10^-4^), melanoma (OR=0.87, 95% CI: 0.79-0.95, *P*=0.002), non-Hodgkin lymphoma (OR=1.29, 95% CI: 1.11-1.49, *P*=6.90×10^-4^), pancreatic cancer (OR=1.50, 95% CI: 1.20-1.87, *P*=4.17×10^-4^) and prostate cancer (OR=0.85, 95% CI: 0.79-0.92, *P*=1.58×10^-5^).

We also identified nominal associations of genetically predicted circulating levels of macrophage inflammatory protein-1β (MIP1β) with the risk of AML (OR=0.86, 95% CI: 0.75-0.99, *P*=0.048), colon cancer (OR=0.95, 95% CI: 0.90-0.99, *P*=0.015) and rectal cancer (OR=0.89, 95% CI: 0.84-0.94, *P*=2.75×10^-5^), as well as eotaxin with the risk of colon cancer (OR=1.28, 95% CI: 1.09-1.49, *P*=0.003), multiple myeloma (OR=1.58, 95% CI: 1.08-2.29, *P*=0.017), and stomach cancer (OR=1.68, 95% CI: 1.16-2.44, *P*=0.006).

## Discussion

In this study, we adopted a two-sample MR approach and systematically evaluated the potential causal effect of 27 circulating cytokines and growth factors on the risk of 20 site-specific cancer. We found that genetically predicted higher levels of IL-18 were associated with an decreased risk of AML, and IL-17 was associated with the risk of stomach cancer. Results were stable in sensitivity analyses using different MR methods and different IV sets. Additionally, we found nominal associations of some cytokine-cancer pairs, suggesting the potential role of these inflammatory biomarkers in the development of certain types of cancer.

IL-18 is a proinflammatory cytokine of the IL-1 family, which can stimulate interferon gamma production and regulate both T helper (Th) 1 and Th2 responses, thus having multiple biological functions (28). IL-18 can promote the proliferation of activated T cells, activation of natural killer cells and cytokine production (29). Protective effects of IL-18 in cancer have been reported in different murine models. For instance, mice receiving IL-18 before or after challenge with CL8-1, both regimens significantly suppressed tumor growth and reduced the number of mice with growth of tumor from 60% (3/5) to 20% (1/5) (30). Hitzler et al. (31) found that IL-18 counteracts IL-1-driven inflammation and limits Helicobacter pathogenic effect. However, observational studies on the association between circulating IL-18 levels and AML are limited to date. A case-control study involving 70 patients and 50 controls reported that there were no differences in the expression level of IL-18 in patients with AML compared to healthy controls (*P*=0.100) (32), while another study of 47 patients with AML found that IL-18 was associated with unfavorable prognostic factors of AML (33). The potential mechanism may be that IL-18 plays an important role in anti-tumor immunity through enhancing interferon-γ production and Fas ligand dependent cytotoxicity of immune cells, and the dose of IL-18 was correlated with the level of serum IFN-γ. Additionally, IL-18 can induce tumor Hapten by activating natural killer (NK) cells, and NK-mediated cytotoxicity of tumor cells, which exert an important role in immune response (30, 34).

As for IL-17, previous observational studies have reported a positive association of circulating IL-17 levels with the risk of stomach cancer. For example, a case-control study including 76 patients and 30 healthy age- and sex-matched controls reported that the median serum levels of IL-17 in patients with stomach cancer were higher than those of controls (9.04 vs. 8.07 pg/ml, *P*=0.010), but serum IL-17 levels were not associated with tumor stage (small (1-2 stage) vs. large (3-4 stage): 9.20 vs. 8.27 pg/ml, *P*=0.410) (35). Another case-control study involving 50 patients and 50 controls reported similar results (36). However, Carneiro et al. reported that IL-17 levels in stomach patients were lower than that in the control group (median: 404.2 vs. 573.9 pg/ml, *P*<0.004) (37). Our MR analysis found an inverse association of circulating IL-17 levels and the risk of stomach cancer. The conflicting results from observational studies may be related to the study design, study population, the stage and type of stomach cancer investigated. For example, a case-control study found that patients with early gastric carcinoma had higher levels of IL-17, whereas individuals with advanced gastric carcinoma had mean IL-17 concentrations comparable to those observed in healthy individuals (38). Another case-only study including 70 patients reported that the expression of IL-17 was lower in patients with diffuse type of stomach cancer compared with those with intestinal type (*P*=0.001) (39). Moreover, Chen et al. found that intratumoral IL-17 expression was an independent factor affecting the five-year overall survival probability in patients with gastric adenocarcinoma (HR: 0.52; 95% CI: 0.33-0.82; *P*=0.005) (40). The potential biological mechanism of IL-17 mediated tumor immunity may be that IL-17 can absorb Th1-related chemokines, such as CXC19 and CXC110, which can promote the migration of effector T cells to tumor sites and increase the number of CD8+ T cells (41).

In addition, we noted nominal cytokine-cancer associations including CTACK and MIP-1β with the risk of several types of cancer. MIP-1β is a member of the chemokine family, and it is related to leukocyte traffic, angiogenesis and metastasis of various types of cancer (42, 43). Observational studies have suggested that the serum concentration of MIP-1β was lower in patients with colorectal cancer as compared to controls (44, 45), which was consistent with our findings. MIP-1β can attract several types of immune cells, including macrophages and T cells. Thus, lower levels of MIP-1β in patients with colon and rectal cancer may be associated with decreased CD 68^+^ tumor-associated macrophage in the invasive margin (44, 46). Similarly, CTACK is a member of the CC chemokine family, which is crucial for the functioning of immune system cells (47). Our study also provided suggestive evidence for potential causal associations of genetically determined circulating levels of CTACK with the risk of prostate, kidney and pancreatic cancer, as well as the risk of melanoma and non-Hodgkin lymphoma. However, observational evidence on CTACK and these types of cancer was limited to date. There was a case-report showing that the pancreatic islets in patients with pancreatic cancer tested positive for CTACK expression (48).

Our study had several limitations. First, our MR estimates may be biased by potential pleiotropy. Therefore, we used different MR approaches and alternative IV sets to assess the influence of pleiotropic SNPs on our MR results. For example, for the association between CTACK and pancreatic cancer, though the MR-Egger regression suggested evidence of pleiotropy (*P* for MR-Egger intercept=0.048), none of the SNPs used as IVs for CTACK have documented associations with other secondary traits, suggesting our results may be biased by unrecognized pleiotropic IVs. Additionally, the GWAS summary statistics used in the present study were all from participants of European ancestry, which may limit the inference of findings to other populations with different ethnicities. Finally, the statistical power may be inadequate in the analyses of certain types of cancer with a limited number of cases, and therefore we may have overlooked potential weak associations.

In conclusion, our findings provide evidence to support potential causal associations of IL-18 with AML and IL-17 with stomach cancer. Further studies are warranted to elucidate underlying biological mechanism and to explore the potential therapeutic targets.

## Data Availability Statement

The original contributions presented in the study are included in the article/**Supplementary Material**. Further inquiries can be directed to the corresponding authors.

## Author Contributions

JS and AL performed the literature review, conducted data analysis, interpreted findings, and drafted the manuscript. LL and BL carried out data analysis and interpreted findings. YQ and DY conducted data analysis, interpreted findings and revised the manuscript. JS, AL and BL took responsibility for the statistical reports, tables and figures of the data analysis. YM and XS directed analytic strategy, supervised the study from conception to completion and revised drafts of the manuscript. All authors read and approved the final version of the manuscript.

## Funding

This work was jointly supported by grants from National Natural Science Foundation of China (82103936, 82174208, and 81973663) and Natural Science Foundation of Zhejiang Province (LQ20H260008 and LQ21H260001). The funder had no role in the study design, data analysis, interpretation of data, or preparation of the manuscript.

## Conflict of Interest

The authors declare that the research was conducted in the absence of any commercial or financial relationships that could be construed as a potential conflict of interest.

## Publisher’s Note

All claims expressed in this article are solely those of the authors and do not necessarily represent those of their affiliated organizations, or those of the publisher, the editors and the reviewers. Any product that may be evaluated in this article, or claim that may be made by its manufacturer, is not guaranteed or endorsed by the publisher.

## Glossary

**Table:**

AML	acute myeloid leukemia
CI	confidence interval
CLL	chronic lymphoid leukemia
CRP	C-reactive protein
CTACK	cutaneous T-cell attracting chemokine
GWAS	genome-wide association study
HR	hazard ratio
IV	instrumental variable
IVW	inverse-variance weighted
IL-17	interleukin-17
IL-18	interleukin-18
MAF	minor allele frequency
MIP1β	macrophage inflammatory protein-1β
MR	Mendelian randomization
OR	odds ratio
PRESSO	Pleiotropy RESidual Sum and Outlier
SD	standard deviation
SNP	single nucleotide polymorphism
TNF-α	tumor necrosis factor α
TNF-γ	tumor necrosis factor γ
TRAIL	tumor necrosis factor related apoptosis-inducing ligand
