# Supplementary material for: Genetically Predicted Circulating Levels of Cytokines and the Risk of Cancer
Source: Front Immunol. 2022 Jul 5;13:886144. doi: 10.3389/fimmu.2022.886144 (PMC9294168; doi:10.3389/fimmu.2022.886144)
Supplement: Supplementary file 1 [file DataSheet_1.docx]

**Supplementary files**

**Table S1**. Detailed information of the studies and datasets used in the present study.

**Table S2**. Genetic instruments of cytokines included in the present study.

**Table S3**. Details of the number of genetic instruments selected for circulating levels of 27 cytokines, variance explained by the selected instruments, and F-statistics for the MR analysis based on the sample size of UKBB.

**Table S4**. Documented pleiotropic associations for the instrumental variables used for circulating IL-17 levels.

**Table S5.** Nominal associations of genetically predicted circulating levels of cytokines with the risk of site-specific cancer based on the inverse variance weighted method.

**Figure S1**. Leave-one-out sensitivity analysis for the association of genetically determined circulating IL-18 levels with the risk of acute myeloid leukemia. The horizontal lines correspond to the odds ratio (OR) and 95% confidence interval (CI) using the inverse variance weighted (IVW) method.

**Table S1. Detailed information of the studies and datasets used in the present study.**

| **Exposure or outcome** | **Study or consortium** | **Participants No. of cases and controls** | | **Web source** |
| --- | --- | --- | --- | --- |
| Cytokines | Ahola-Olli et al, 2017 | 8,293 Finns / | | [http://computationalmedicine.fi/data#Cytokine_GWAS](#Cytokine_GWAS) |
| 20 site-specific cancer types | UK Biobank | 456,348 unrelated European-descent individuals | Acute myeloid leukemia (312 cases and 456,036 controls) | <https://www.ukbiobank.ac.uk/> |
|  |  |  | Bladder cancer (2,264 cases and 454,084 controls) |  |
|  |  |  | Brain caner (497 cases and 455,851 controls) |  |
|  |  |  | Breast cancer (10,971 cases and 445,377 controls) |  |
|  |  |  | Chronic lymphoid leukemia (356 cases and 455,992 controls) |  |
|  |  |  | Colon caner (3,221 cases and 453,127 controls) |  |
|  |  |  | Esophagus cancer (750 cases and 455,598 controls) |  |
|  |  |  | Kidney cancer (988 cases and 455,360 controls) |  |
|  |  |  | Liver cancer (214 cases and 456,134 controls) |  |
|  |  |  | Lung cancer (2,120 cases and 454,228 controls) |  |
|  |  |  | Melanoma (3,564 cases and 452,712 controls) |  |
|  |  |  | Multiple myeloma (564 cases and 455,784 controls) |  |
|  |  |  | Non-Hodgkin lymphoma (1,395 cases and 454,953 controls) |  |
|  |  |  | Ovarian cancer (779 cases and 246,729 controls) |  |
|  |  |  | Pancreatic cancer (587 cases and 455,761 controls) |  |
|  |  |  | Prostate cancer (5,796 cases and 203,012 controls) |  |
|  |  |  | Rectal cancer (2,202 cases and 454,146 controls) |  |
|  |  |  | Stomach cancer (569 cases and 455,779 controls) |  |
|  |  |  | Thyroid cancer (379 cases and 455,969 controls) |  |
|  |  |  | Uterus cancer (1,388 cases and 246,152 controls) |  |

**Table S2. Genetic instruments of cytokines included in the present study.**

| **Cytokines** | **Abbreviation** | **No. of independent genome-wide significant SNPs** | **No. of SNPs included in**  **the analysis** |
| --- | --- | --- | --- |
| Beta nerve growth factor | β-NGF | 2 | 1 |
| Cutaneous T-cell attracting (CCL27) | CTACK | 21 | 7 |
| Eotaxin (CCL11) | Eotaxin | 19 | 5 |
| Basic fibroblast growth factor | FGF-basic | 0 | 0 |
| Granulocyte colony-stimulating factor | G-CSF | 0 | 0 |
| Growth regulated oncogene-α (CXCL1) | GRO-α | 32 | 4 |
| Hepatocyte growth factor | HGF | 7 | 2 |
| Interferon-gamma | IFN-γ | 1 | 1 |
| Interleukin-1 receptor antagonist | IL-1rα | 0 | 0 |
| Interleukin-1-beta | IL-1β | 0 | 0 |
| Interleukin-2 | IL-2 | 0 | 0 |
| Interleukin-2 receptor, alpha subunit | IL-2rα | 16 | 3 |
| Interleukin-4 | IL-4 | 0 | 0 |
| Interleukin-5 | IL-5 | 0 | 0 |
| Interleukin-6 | IL-6 | 0 | 0 |
| Interleukin-7 | IL-7 | 2 | 1 |
| Interleukin-8 (CXCL8) | IL-8 | 0 | 0 |
| Interleukin-9 | IL-9 | 0 | 0 |
| Interleukin-10 | IL-10 | 1 | 1 |
| Interleukin-12p70 | IL-12p70 | 9 | 4 |
| Interleukin-13 | IL-13 | 1 | 1 |
| Interleukin-16 | IL-16 | 7 | 3 |
| Interleukin-17 | IL-17 | 2 | 2 |
| Interleukin-18 | IL-18 | 90 | 16 |
| Interferon gamma-induced protein 10 (CXCL10) | IP-10 | 3 | 3 |
| Macrophage colony-stimulating factor | M-CSF | 1 | 1 |
| Monocyte chemotactic protein-1 (CCL2) | MCP-1 | 41 | 13 |
| Monocyte specific chemokine 3 (CCL7) | MCP-3 | 0 | 0 |
| Macrophage migration inhibitory factor (glycosylation-inhibiting factor) | MIF | 2 | 1 |
| Monokine induced by interferon-gamma (CXCL9) | MIG | 5 | 1 |
| Macrophage inflammatory protein-1α (CCL3) | MIP-1α | 0 | 0 |
| Macrophage inflammatory protein-1β (CCL4) | MIP-1β | 393 | 90 |
| Platelet derived growth factor BB | PDGF-bb | 30 | 10 |
| Regulated on activation, normal T Cell expressed and secreted (CCL5) | RANTES | 1 | 1 |
| Stem cell factor | SCF | 2 | 2 |
| Stem cell growth factor beta | SCGF-β | 29 | 7 |
| Stromal cell-derived factor-1 alpha (CXCL12) | SDF-1α | 0 | 0 |
| Tumor necrosis factor-alpha | TNF-α | 0 | 0 |
| Tumor necrosis factor-beta | TNF-β | 3 | 2 |
| TNF-related apoptosis inducing ligand | TRAIL | 92 | 32 |
| Vascular endothelial growth factor | VEGF | 17 | 10 |

Abbreviation: SNP, single nucleotide polymorphism.

**Table S3. Details of the number of genetic instruments selected for circulating levels of 27 cytokines, variance explained by the selected instruments, and F-statistics for the MR analysis based on the sample size of UKBB.**

| **Cytokines** | **No. of SNPs included in the analysis** | **Variance explained (R^2^)^*^** | **F-statistic, median**  **(range)^¶^** |
| --- | --- | --- | --- |
| β-NGF | 1 | 0.011 | 36.50 |
| CTACK | 7 | 0.143 | 80.58 (41.59-142.66) |
| Eotaxin | 5 | 0.026 | 44.04 (37.76-203.26) |
| GRO-α | 4 | 0.213 | 64.68 (30.28-184.38) |
| HGF | 2 | 0.009 | 49.03 (40.82-57.25) |
| IFN-γ | 1 | 0.001 | 32.34 |
| IL-2rα | 3 | 0.075 | 103.12 (36.59-167.61) |
| IL-7 | 1 | 0.052 | 169.84 |
| IL-10 | 1 | 0.005 | 37.50 |
| IL-12p70 | 4 | 0.021 | 39.52 (33.76-44.31) |
| IL-13 | 1 | 0.084 | 292.85 |
| IL-16 | 3 | 0.163 | 40.59 (31.06-131.98) |
| IL-17 | 2 | 0.010 | 35.09 (31.21-38.97) |
| IL-18 | 16 | 0.109 | 57.37 (31.64-96.14) |
| IP-10 | 3 | 0.045 | 32.04 (31.11-62.35) |
| M-CSF | 1 | 0.007 | 31.64 |
| MCP-1 | 13 | 0.067 | 38.29 (30.32-91.76) |
| MIF | 1 | 0.012 | 39.05 |
| MIG | 1 | 0.009 | 42.38 |
| MIP-1β | 90 | 0.811 | 48.30 (29.79-788.96) |
| PDGF-bb | 10 | 0.065 | 44.46 (30.83-245.35) |
| RANTES | 1 | 0.002 | 30.00 |
| SCF | 2 | 0.011 | 40.24 (31.80-48.67) |
| SCGF-β | 7 | 0.071 | 61.36 (35.19-67.85) |
| TNF-β | 2 | 0.115 | 81.5 (39.62-123.38) |
| TRAIL | 32 | 0.340 | 89.26 (32.06-648.03) |
| VEGF | 10 | 0.051 | 39.03 (30.85-62.53) |

*$\text{ }\text{R}^{\text{2}}\text{= }\text{2×}\text{β}^{\text{2}}\text{×MAF×(1-MAF)/}\text{(}\text{2 ×}\text{β}^{\text{2}}\text{×MAF×(1-MAF}\text{)}\text{+}{\text{(SE}\left( \text{β} \right)\text{)}}^{\text{2}}\text{×2×N×MAF×(1-MAF))}$. β: effect size estimates of the SNPs of cytokines levels; MAF: minimum allele frequency. The variance of each cytokine was calculated by using an additive model under the assumption of no interaction between each SNPs. (Yarmolinsky J *et al*, J Natl Cancer Inst, 2018;110(9):1035-1038.)

**¶** $\text{F=}\text{R}^{\text{2}}\text{×(N-1-k))/((1-}\text{R}^{\text{2}}\text{)×k)}$. R^2^ represents the variance explained by the IVs; N is the sample size; k is the number of SNPs included in the instrument. (Bowden J *et al*, Genet Epidemiol, 2016;40(4):304-14).

| **Table S4. Documented pleiotropic associations for the instrumental variables used for circulating IL-17 levels.** | | | | |
| --- | --- | --- | --- | --- |
| **SNP** | **Trait(s)** | ***P*-value** | **PubMed** |  |
| rs1530455 | Mean platelet volume | 3.11×10^-29^ | 27863252 |  |
|  | Platelet count | 7.39×10^-25^ | 27863252 |  |
|  | Platelet distribution width | 5.05×10^-31^ | 27863252 |  |
|  | Plateletcrit | 2.22×10^-9^ | 27863252 |  |

Abbreviations: IL-17, Interleukin-17; SNP, single nucleotide polymorphism.

**Table S5. Nominal associations of genetically predicted circulating levels of cytokines with the risk of site-specific cancer based on the inverse variance weighted method.**

| **Outcomes and exposures** | **No. of SNPs** | **OR (95% CI)** | **P for association** | ***P* for heterogeneity** | **P intercept from MR-Egger regression** |
| --- | --- | --- | --- | --- | --- |
| **Acute myeloid leukemia** |  |  |  |  |  |
| GRO-α |  |  |  |  |  |
| Inverse-variance weighted | 4 | 0.68 (0.51, 0.93) | 0.014 | 0.895 |  |
| Weighted median | 4 | 0.68 (0.48, 0.97) | 0.033 |  |  |
| Maximum-likelihood | 4 | 0.68 (0.50, 0.93) | 0.015 |  |  |
| MR-PRESSO | 4 | 0.68 (0.60, 0.78) | 0.012 |  |  |
| MR-Egger | 4 | / | / |  | 0.644 |
| MIF |  |  |  |  |  |
| Inverse-variance weighted | 1 | 0.32 (0.11, 0.90) | 0.031 |  |  |
| MIP-1β |  |  |  |  |  |
| Inverse-variance weighted | 90 | 0.86 (0.75, 0.99) | 0.048 | 0.576 |  |
| Weighted median | 90 | 0.92 (0.74, 1.15) | 0.474 |  |  |
| Maximum-likelihood | 90 | 0.86 (0.75, 1.00) | 0.050 |  |  |
| MR-PRESSO | 90 | 0.86 (0.75, 0.99) | 0.042 |  |  |
| MR-Egger | 90 | / | / |  | 0.281 |
| TRAIL |  |  |  |  |  |
| Inverse-variance weighted | 32 | 1.27 (1.06, 1.52) | 0.011 | 0.431 |  |
| Weighted median | 32 | 1.39 (1.08, 1.79) | 0.012 |  |  |
| Maximum-likelihood | 32 | 1.27 (1.06, 1.52) | 0.011 |  |  |
| MR-PRESSO | 32 | 1.27 (1.05, 1.52) | 0.017 |  |  |
| MR-Egger | 32 | / | / |  | 0.156 |
| **Bladder cancer** |  |  |  |  |  |
| IL-18 |  |  |  |  |  |
| Inverse-variance weighted | 16 | 0.86 (0.79, 0.93) | 3.30×10^-4^ | 0.961 |  |
| Weighted median | 16 | 0.84 (0.76, 0.94) | 0.003 |  |  |
| Maximum-likelihood | 16 | 0.86 (0.79, 0.93) | 3.79×10^-4^ |  |  |
| MR-PRESSO | 16 | 0.86 (0.81, 0.91) | 3.71×10^-4^ |  |  |
| MR-Egger | 16 | / | / |  | 0.240 |
| SCGF-β |  |  |  |  |  |
| Inverse-variance weighted | 7 | 1.18 (1.01, 1.38) | 0.038 | 0.692 |  |
| Weighted median | 7 | 1.25 (1.03, 1.52) | 0.099 |  |  |
| Maximum-likelihood | 7 | 1.18 (1.01, 1.39) | 0.038 |  |  |
| MR-PRESSO | 7 | 1.18 (1.04, 1.34) | 0.042 |  |  |
| MR-Egger | 7 | / | / |  | 0.647 |
| **Breast cancer** |  |  |  |  |  |
| IL-18 |  |  |  |  |  |
| Inverse-variance weighted | 16 | 1.05 (1.01, 1.09) | 0.027 | 0.940 |  |
| Weighted median | 16 | 1.05 (1.00, 1.10) | 0.076 |  |  |
| Maximum-likelihood | 16 | 1.05 (1.01, 1.09) | 0.015 |  |  |
| MR-PRESSO | 16 | 1.05 (1.02, 1.08) | 0.004 |  |  |
| MR-Egger | 16 | / | / |  | 0.756 |
| **Chronic lymphoid leukemia** |  |  |  |  |  |
| GRO-α |  |  |  |  |  |
| Inverse-variance weighted | 4 | 0.74 (0.56, 0.99) | 0.041 | 0.894 |  |
| Weighted median | 4 | 0.73 (0.53, 1.02) | 0.063 |  |  |
| Maximum-likelihood | 4 | 0.74 (0.56, 0.99) | 0.042 |  |  |
| MR-PRESSO | 4 | 0.74 (0.65, 0.85) | 0.020 |  |  |
| MR-Egger | 4 | / | / |  | 0.565 |
| HGF |  |  |  |  |  |
| Inverse-variance weighted | 2 | 0.37 (0.17, 0.83) | 0.015 | 0.957 |  |
| Maximum-likelihood | 2 | 0.37 (0.16, 0.85) | 0.018 |  |  |
| IL-18 |  |  |  |  |  |
| Inverse-variance weighted | 16 | 1.39 (1.29, 1.71) | 0.002 | 0.774 |  |
| Weighted median | 16 | 1.52 (1.14, 2.02) | 0.004 |  |  |
| Maximum-likelihood | 16 | 1.40 (1.13, 1.73) | 0.002 |  |  |
| MR-PRESSO | 16 | 1.39 (1.17, 1.66) | 0.002 |  |  |
| MR-Egger | 16 | / | / |  | 0.067 |
| TRAIL |  |  |  |  |  |
| Inverse-variance weighted | 32 | 1.27 (1.06, 1.52) | 0.011 | 0.431 |  |
| Weighted median | 32 | 1.08 (0.77, 1.79) | 0.011 |  |  |
| Maximum-likelihood | 32 | 1.27 (1.05, 1.52) | 0.011 |  |  |
| MR-PRESSO | 32 | 1.27 (1.05, 1.52) | 0.017 |  |  |
| MR-Egger | 32 | / | / |  | 0.156 |
| **Colon cancer** |  |  |  |  |  |
| Eotain |  |  |  |  |  |
| Inverse-variance weighted | 5 | 1.28 (1.09, 1.49) | 0.003 | 0.334 |  |
| Weighted median | 5 | 1.25 (1.04, 1.51) | 0.018 |  |  |
| Maximum-likelihood | 5 | 1.28 (1.08, 1.51) | 0.004 |  |  |
| MR-PRESSO | 5 | 1.27 (1.08, 1.51) | 0.005 |  |  |
| MR-Egger | 5 | / | / |  | 0.231 |
| MIP-1β |  |  |  |  |  |
| Inverse-variance weighted | 90 | 0.95 (0.90, 0.99) | 0.015 | 0.526 |  |
| Weighted median | 90 | 0.93 (0.86, 1.00) | 0.051 |  |  |
| Maximum-likelihood | 90 | 0.95 (0.90, 0.99) | 0.017 |  |  |
| MR-PRESSO | 90 | 0.95 (0.90, 0.99) | 0.017 |  |  |
| MR-Egger | 90 | / | / |  | 0.785 |
| RANTES |  |  |  |  |  |
| Inverse-variance weighted | 1 | 0.53 (0.34, 0.87) | 0.010 |  |  |
| TRAIL |  |  |  |  |  |
| Inverse-variance weighted | 32 | 1.07 (1.01, 1.13) | 0.021 | 0.980 |  |
| Weighted median | 32 | 1.06 (0.98, 1.15) | 0.137 |  |  |
| Maximum-likelihood | 32 | 1.07 (1.01, 1.13) | 0.021 |  |  |
| MR-PRESSO | 32 | 1.07 (1.02, 1.11) | 0.021 |  |  |
| MR-Egger | 32 | / | / |  | 0.470 |
| **Esophagus cancer** |  |  |  |  |  |
| IFN-γ |  |  |  |  |  |
| Inverse-variance weighted | 1 | 2.44 (1.40, 4.26) | 0.002 |  |  |
| **Kidney cancer** |  |  |  |  |  |
| CTACK |  |  |  |  |  |
| Inverse-variance weighted | 7 | 0.72 (0.64, 0.85) | 1.64×10^-4^ | 0.952 |  |
| Weighted median | 7 | 0.74 (0.60, 0.91) | 4.06×10^-3^ |  |  |
| Maximum-likelihood | 7 | 0.72 (0.60, 0.86) | 2.06×10^-4^ |  |  |
| MR-PRESSO | 7 | 0.72 (0.66, 0.78) | 3.41×10^-4^ |  |  |
| MR-Egger | 7 | / | / |  | 0.933 |
| IL-16 |  |  |  |  |  |
| Inverse-variance weighted | 3 | 0.80 (0.66, 0.97) | 0.020 | 0.799 |  |
| Weighted median | 3 | 0.79 (0.65, 0.97) | 0.022 |  |  |
| Maximum-likelihood | 3 | 0.80 (0.66, 0.97) | 0.021 |  |  |
| MR-Egger | 3 | / | / |  | 0.537 |
| IL-18 |  |  |  |  |  |
| Inverse-variance weighted | 16 | 0.87 (0.77, 0.99) | 0.028 | 0.999 |  |
| Weighted median | 16 | 0.88 (0.75, 1.04) | 0.141 |  |  |
| Maximum-likelihood | 16 | 0.87 (0.77, 0.99) | 0.028 |  |  |
| MR-PRESSO | 16 | 0.87 (0.82, 0.92) | 0.001 |  |  |
| MR-Egger | 16 | / | / |  | 0.834 |
| IP-10 |  |  |  |  |  |
| Inverse-variance weighted | 3 | 0.68 (0.47, 0.98) | 0.039 | 0.195 |  |
| Weighted median | 3 | 0.56 (0.35, 0.88) | 0.012 |  |  |
| Maximum-likelihood | 3 | 0.87 (0.76, 0.99) | 0.040 |  |  |
| MR-Egger | 3 | / | / |  | 0.224 |
| **Lung cancer** |  |  |  |  |  |
| TRAIL |  |  |  |  |  |
| Inverse-variance weighted | 32 | 0.93 (0.87, 0.99) | 0.034 | 0.398 |  |
| Weighted median | 32 | 0.93 (0.84, 1.04) | 0.199 |  |  |
| Maximum-likelihood | 32 | 0.93 (0.86, 0.99) | 0.030 |  |  |
| MR-PRESSO | 32 | 0.93 (0.86, 0.99) | 0.047 |  |  |
| MR-Egger | 32 | / | / |  | 0.925 |
| **Melanoma** |  |  |  |  |  |
| CTACK |  |  |  |  |  |
| Inverse-variance weighted | 7 | 0.87 (0.79, 0.95) | 0.002 | 0.518 |  |
| Weighted median | 7 | 0.85 (0.75, 0.96) | 0.006 |  |  |
| Maximum-likelihood | 7 | 0.87 (0.79, 0.95) | 0.002 |  |  |
| MR-PRESSO | 7 | 0.87 (0.80, 0.94) | 0.016 |  |  |
| MR-Egger | 7 | / | / |  | 0.177 |
| IL-12p70 |  |  |  |  |  |
| Inverse-variance weighted | 4 | 1.39 (1.11, 1.73) | 0.004 | 0.855 |  |
| Weighted median | 4 | 1.38 (1.06, 1.80) | 0.019 |  |  |
| Maximum-likelihood | 4 | 1.39 (1.11, 1.74) | 0.004 |  |  |
| MR-PRESSO | 4 | 1.38 (1.24, 1.55) | 0.011 |  |  |
| MR-Egger | 4 | / | / |  | 0.730 |
| IL-16 |  |  |  |  |  |
| Inverse-variance weighted | 3 | 1.11 (1.01, 1.23) | 0.037 | 0.946 |  |
| Weighted median | 3 | 1.11 (1.00, 1.24) | 0.043 |  |  |
| Maximum-likelihood | 3 | 1.11 (1.01, 1.23) | 0.039 |  |  |
| MR-Egger | 3 | / | / |  | 0.742 |
| MIF |  |  |  |  |  |
| Inverse-variance weighted | 1 | 1.75 (1.29, 2.38) | 3.64×10-4 |  |  |
| **Multiple myeloma** |  |  |  |  |  |
| Eotaxin |  |  |  |  |  |
| Inverse-variance weighted | 5 | 1.58 (1.08, 2.29) | 0.017 | 0.069 |  |
| Weighted median | 5 | 1.85 (1.10, 3.11) | 0.021 |  |  |
| Maximum-likelihood | 5 | 1.59 (0.91, 2.78) | 0.104 |  |  |
| MR-PRESSO | 5 | 1.58 (0.91, 2.74) | 0.182 |  |  |
| MR-Egger | 5 | / | / |  | 0.277 |
| IL-18 |  |  |  |  |  |
| Inverse-variance weighted | 16 | 0.76 (0.63, 0.90) | 0.002 | 0.310 |  |
| Weighted median | 16 | 0.66 (0.52, 0.84) | 0.001 |  |  |
| Maximum-likelihood | 16 | 0.76 (0.64, 0.91) | 0.003 |  |  |
| MR-PRESSO | 16 | 0.76 (0.63, 0.90) | 0.007 |  |  |
| MR-Egger | 16 | / | / |  | 0.125 |
| MCP-1 |  |  |  |  |  |
| Inverse-variance weighted | 13 | 1.40 (1.02, 1.93) | 0.039 | 0.908 |  |
| Weighted median | 13 | 1.93 (0.78, 1.82) | 0.414 |  |  |
| Maximum-likelihood | 13 | 1.41 (1.02, 1.95) | 0.036 |  |  |
| MR-PRESSO | 13 | 1.40 (1.11, 1.76) | 0.016 |  |  |
| MR-Egger | 13 | / | / |  | 0.792 |
| **Non-Hodgkin lymphoma** |  |  |  |  |  |
| CTACK |  |  |  |  |  |
| Inverse-variance weighted | 7 | 1.29 (1.11, 1.49) | 6.90×10^-4^ | 0.977 |  |
| Weighted median | 7 | 1.30 (1.09, 1.56) | 4.13×10^-3^ |  |  |
| Maximum-likelihood | 7 | 1.29 (1.11, 1.49) | 7.82×10^-4^ |  |  |
| MR-PRESSO | 7 | 1.29 (1.21, 1.37) | 2.68×10^-4^ |  |  |
| MR-Egger | 7 | / | / |  | 0.539 |
| IL-17 |  |  |  |  |  |
| Inverse-variance weighted | 2 | 1.83 (1.07, 3.13) | 0.028 | 0.803 |  |
| Maximum-likelihood | 2 | 1.83 (1.05, 3.19) | 0.033 |  |  |
| IL-18 |  |  |  |  |  |
| Inverse-variance weighted | 16 | 0.84 (0.74, 0.96) | 0.008 | 0.094 |  |
| Weighted median | 16 | 0.77 (0.66, 0.89) | 0.001 |  |  |
| Maximum-likelihood | 16 | 0.84 (0.74, 0.96) | 0.010 |  |  |
| MR-PRESSO | 16 | 0.84 (0.74, 0.96) | 0.007 |  |  |
| MR-Egger | 16 | / | / |  | 0.043 |
| TRAIL |  |  |  |  |  |
| Inverse-variance weighted | 32 | 0.91 (0.84, 0.99) | 0.034 | 0.933 |  |
| Weighted median | 32 | 0.90 (0.79, 1.02) | 0.092 |  |  |
| Maximum-likelihood | 32 | 0.91 (0.84, 0.99) | 0.034 |  |  |
| MR-PRESSO | 32 | 0.91 (0.85, 0.98) | 0.013 |  |  |
| MR-Egger | 32 | / | / |  | 0.979 |
| **Ovary cancer** |  |  |  |  |  |
| β-NGF |  |  |  |  |  |
| Inverse-variance weighted | 1 | 2.01 (1.05, 3.86) | 0.350 |  |  |
| SCGF-β |  |  |  |  |  |
| Inverse-variance weighted | 7 | 0.72 (0.55, 0.93) | 0.014 | 0.166 |  |
| Weighted median | 7 | 0.60 (0.42, 0.85) | 0.004 |  |  |
| Maximum-likelihood | 7 | 0.71 (0.51, 0.99) | 0.043 |  |  |
| MR-PRESSO | 7 | 0.72 (0.51, 0.99) | 0.046 |  |  |
| MR-Egger | 7 | / | / |  | 0.944 |
| **Pancreatic cancer** |  |  |  |  |  |
| CTACK |  |  |  |  |  |
| Inverse-variance weighted | 7 | 1.50 (1.20, 1.87) | 4.17×10^-4^ | 0.637 |  |
| Weighted median | 7 | 1.65 (1.23, 2.22) | 8.05×10^-4^ |  |  |
| Maximum-likelihood | 7 | 1.50 (1.20, 1.89) | 4.67×10^-4^ |  |  |
| MR-PRESSO | 7 | 1.50 (1.24, 1.80) | 5.97×10^-3^ |  |  |
| MR-Egger | 7 | / | / |  | 0.048 |
| **Prostate cancer** |  |  |  |  |  |
| CTACK |  |  |  |  |  |
| Inverse-variance weighted | 7 | 0.85 (0.79, 0.92) | 1.58×10^-5^ | 0.240 |  |
| Weighted median | 7 | 0.81 (0.74, 0.90) | 4.38×10^-5^ |  |  |
| Maximum-likelihood | 7 | 0.85 (0.78, 0.92) | 1.56×10^-4^ |  |  |
| MR-PRESSO | 7 | 0.85 (0.78, 0.93) | 9.55×10^-3^ |  |  |
| MR-Egger | 7 | / | / |  | 0.008 |
| IL-7 |  |  |  |  |  |
| Inverse-variance weighted | 1 | 1.13 (1.00, 1.27) | 0.046 |  |  |
| IL-13 |  |  |  |  |  |
| Inverse-variance weighted | 1 | 1.10 (1.00, 1.20) | 0.049 |  |  |
| MIF |  |  |  |  |  |
| Inverse-variance weighted | 1 | 0.70 (0.55, 0.90) | 0.005 |  |  |
| MIG |  |  |  |  |  |
| Inverse-variance weighted | 1 | 1.36 (01.04, 1.76) | 0.023 |  |  |
| **Rectal cancer** |  |  |  |  |  |
| MIP-1β |  |  |  |  |  |
| Inverse-variance weighted | 90 | 0.89 (0.84, 0.94) | 2.75×10^-5^ | 0.094 |  |
| Weighted median | 90 | 0.95 (0.87, 1.04) | 0.250 |  |  |
| Maximum-likelihood | 90 | 0.89 (0.84, 0.94) | 2.05×10^-4^ |  |  |
| MR-PRESSO | 90 | 0.89 (0.84, 0.95) | 1.39×10^-4^ |  |  |
| MR-Egger | 90 | / | / |  | 0.635 |
| PDHF-bb |  |  |  |  |  |
| Inverse-variance weighted | 10 | 0.86 (0.74, 0.99) | 0.043 | 0.913 |  |
| Weighted median | 10 | 0.88 (0.73, 1.06) | 0.167 |  |  |
| Maximum-likelihood | 10 | 0.86 (0.74, 0.99) | 0.044 |  |  |
| MR-PRESSO | 10 | 0.86 (0.78, 0.95) | 0.014 |  |  |
| MR-Egger | 10 | / | / |  | 0.662 |
| TRAIL |  |  |  |  |  |
| Inverse-variance weighted | 32 | 1.08 (1.01, 1.15) | 0.032 | 0.352 |  |
| Weighted median | 32 | 1.07 (0.96, 1.18) | 0.211 |  |  |
| Maximum-likelihood | 32 | 1.08 (1.01, 1.16) | 0.036 |  |  |
| MR-PRESSO | 32 | 1.08 (1.00, 1.16) | 0.047 |  |  |
| MR-Egger | 32 | / | / |  | 0.221 |
| **Stomach cancer** |  |  |  |  |  |
| Eotaxin |  |  |  |  |  |
| Inverse-variance weighted | 5 | 1.68 (1.16, 2.44) | 0.006 | 0.322 |  |
| Weighted median | 5 | 1.39 (0.88, 2.19) | 0.161 |  |  |
| Maximum-likelihood | 5 | 1.69 (1.13, 2.53) | 0.011 |  |  |
| MR-PRESSO | 5 | 1.68 (1.12, 2.52) | 0.011 |  |  |
| MR-Egger | 5 | / | / |  | 0.170 |
| IFN-γ |  |  |  |  |  |
| Inverse-variance weighted | 1 | 0.16 (0.04, 0.66) | 0.011 |  |  |
| **Thyroid cancer** |  |  |  |  |  |
| IL-17 |  |  |  |  |  |
| Inverse-variance weighted | 2 | 0.33 (0.12, 0.93) | 0.036 | 0.905 |  |
| Maximum-likelihood | 2 | 0.33 (0.12, 0.96) | 0.042 |  |  |
| MCP-1 |  |  |  |  |  |
| Inverse-variance weighted | 13 | 0.62 (0.42, 0.92) | 0.016 | 0.841 |  |
| Weighted median | 13 | 0.61 (0.36, 1.03) | 0.066 |  |  |
| Maximum-likelihood | 13 | 0.62 (0.42, 0.92) | 0.018 |  |  |
| MR-PRESSO | 13 | 0.62 (0.46, 0.84) | 0.009 |  |  |
| MR-Egger | 13 | / | / |  | 0.432 |
| **Uterus cancer** |  |  |  |  |  |
| TNF-β |  |  |  |  |  |
| Inverse-variance weighted | 1 | 0.77 (0.62, 0.96) | 0.021 |  |  |

Abbreviations: CI, confidence interval; IVW, inverse-variance weighted; MR-PRESSO, Mendelian randomization pleiotropy residual sum and outlier; OR, odds ratio; SNP, single nucleotide polymorphism.

**
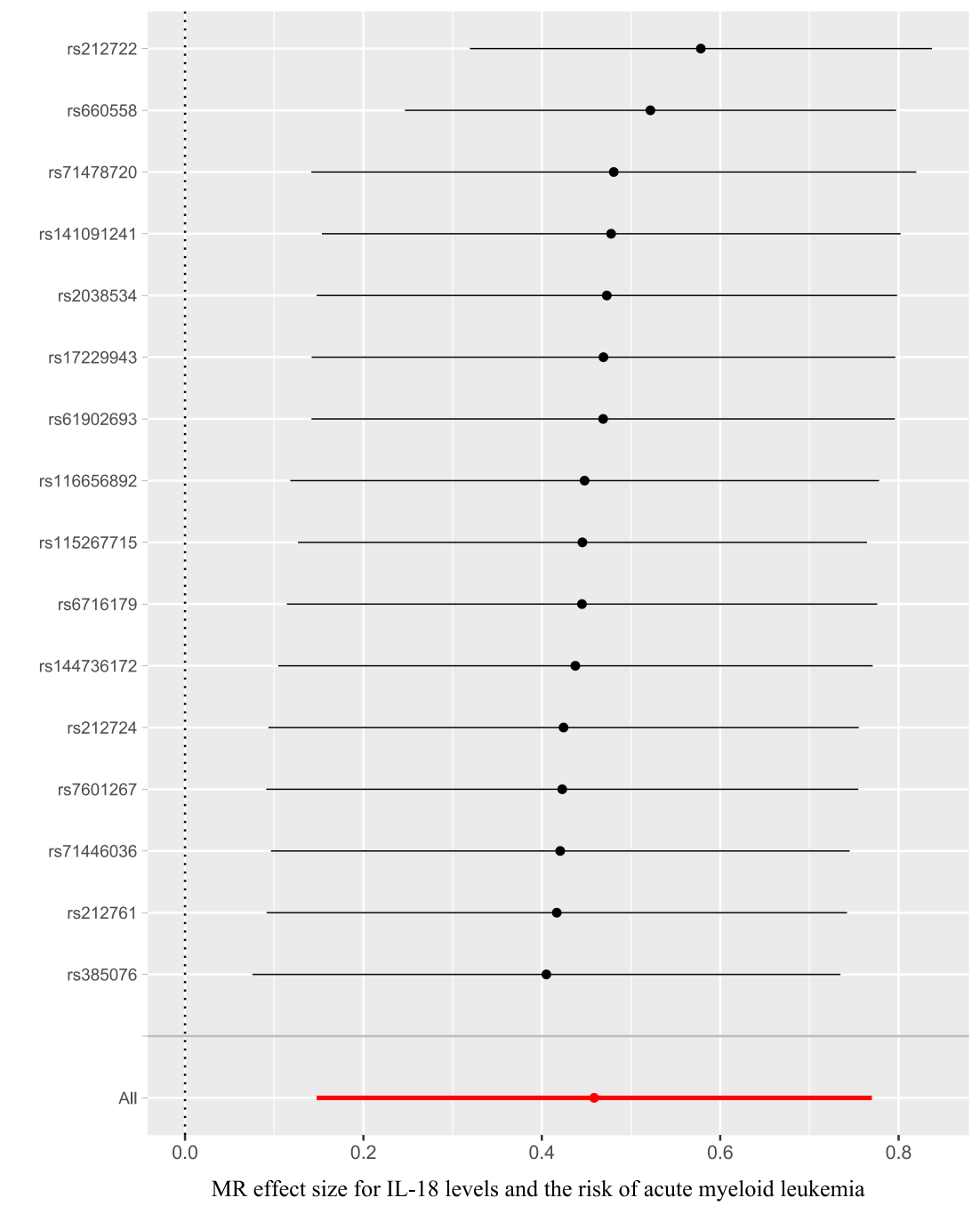
**

**Figure S1**. Leave-one-out sensitivity analysis for the association of genetically determined circulating IL-18 levels with the risk of acute myeloid leukemia. The horizontal lines correspond to the odds ratio (OR) and 95% confidence interval (CI) using the inverse variance weighted (IVW) method.
